# The ontology-based answers (OBA) service: a connector for embedded usage of ontologies in applications

**DOI:** 10.3389/fgene.2012.00197

**Published:** 2012-10-05

**Authors:** Jürgen Dönitz, Edgar Wingender

**Affiliations:** ^1^Department of Bioinformatics, University Medical Center GöttingenGöttingen, Germany; ^2^Department of Developmental Biology, Johann-Friedrich-Blumenbach Institute for Zoology and Anthropology, Georg-August University GöttingenGöttingen, Germany

**Keywords:** ontology, semantic function, ontology-based answers, OBA

## Abstract

The semantic web depends on the use of ontologies to let electronic systems interpret contextual information. Optimally, the handling and access of ontologies should be completely transparent to the user. As a means to this end, we have developed a service that attempts to bridge the gap between experts in a certain knowledge domain, ontologists, and application developers. The ontology-based answers (OBA) service introduced here can be embedded into custom applications to grant access to the classes of ontologies and their relations as most important structural features as well as to information encoded in the relations between ontology classes. Thus computational biologists can benefit from ontologies without detailed knowledge about the respective ontology. The content of ontologies is mapped to a graph of connected objects which is compatible to the object-oriented programming style in Java. Semantic functions implement knowledge about the complex semantics of an ontology beyond the class hierarchy and “partOf” relations. By using these OBA functions an application can, for example, provide a semantic search function, or (in the examples outlined) map an anatomical structure to the organs it belongs to. The semantic functions relieve the application developer from the necessity of acquiring in-depth knowledge about the semantics and curation guidelines of the used ontologies by implementing the required knowledge. The architecture of the OBA service encapsulates the logic to process ontologies in order to achieve a separation from the application logic. A public server with the current plugins is available and can be used with the provided connector in a custom application in scenarios analogous to the presented use cases. The server and the client are freely available if a project requires the use of custom plugins or non-public ontologies. The OBA service and further documentation is available at http://www.bioinf.med.uni-goettingen.de/projects/oba

## INTRODUCTION

Ontologies play a major role in the semantic web (Berners-Lee et al., 2001). Running in the background they provide electronic systems with the expertise of a knowledge domain. Through formal and logical statements ontologies are useful to unambiguously identify and define entities representing material objects as well as abstract concepts and their mutual relations. By connecting unknown terms with known ones through defined statements, new knowledge can be deduced. This knowledge can be used to provide the user with information that he/she is seeking but could not exactly specify. This is achieved by means of a mandatory class hierarchy, using the “is_a” relation, and other relations, connecting the ontology classes to each other. Supplementary data can be added to each ontology class by annotations. While the meaning of relations is comprehensible to human users so that they can select the right one for traversing the graph, it is a particular challenge to transfer the logical axioms defined in an ontology into an object-oriented view that is common to most applications (Winston et al., 1987; Burger et al., 2008).

A multitude of tools and web services dealing with ontologies are available in the biomedical field. Ontology browsers like Amigo (Carbon et al., 2009) for the Gene Ontology (GO; Ashburner et al., 2000) or ontology editors (OBOEdit: Day-Richter et al., 2007; Protégé^1^) let the user work interactively with an ontology. The web services Ontology Lookup Service (OLS; Côté et al., 2008), the NCBO BioPortal (Noy et al., 2009) and OntoCAT (Adamusiak et al., 2011) facilitate the search function covering all ontologies publicly available at the NCBO portal or the OBO-Foundry (Smith et al., 2007) and provide access to their content. OntoCAT and the BioPortal also offer an interface to be queried by electronic systems over the network. By doing so OntoCAT additionally offers a Java and R client (Kurbatova et al., 2011) for communication with the service.

The listed portals offer services which are highly valuable to the community. However, they fall short in two aspects: by approaching the access of a collection of ontologies in a standardized way, the portals lack functions that are specific for individual ontologies, leaving the information encoded in the diverse relationships unattended. An automated system does not allow the user to decide when to use which relationship, the algorithm has to solve this problem. The application developer is required to be familiar with the annotation guidelines and implement the required algorithm.

If a search interface allows the user to enter or select an anatomical structure, for which data should be displayed, the user will expect results not only for the selected structures, but also for substructures and perhaps functionally related structures. With the use of ontologies this challenge can be met. The different sets of available relations used in ontologies like “part_of,” “contained,” or “bordered_by” require an implementation of such a search algorithm to be ontology specific.

The other challenge is between the semantics of ontologies, consisting of a set of axioms, and the modern style of object-oriented programming. In an ontology the classes and their relations are stored in separate axioms while in an object graph the objects themselves have knowledge about the links to their neighbors. APIs like OWL-API (Horridge and Bechhofer, 2011) or Jena-API (Jena – A Semantic Web Framework for Java^2^) facilitate full access to ontologies and follow their design principles. They disclose any information and logic of the supported ontology format to the user. The resulting complexity prevents a straight way to get, e.g., neighbors of a class from the ontology. To get the subclasses of an ontology class with the OWL-API the axioms for the superclass has to be fetched and the right axioms have to be selected. Also when using the ontology portals an additional request to the portal is required because the ontology classes fetched from the portals lack a method to access their own subclasses.

As an alternative way we suggest a service providing ontology-based answers (OBA service). To benefit from ontologies the OBA service can be embedded in applications and workflows. The OBA project’s goal is to make knowledge, which a user can intuitively retrieve from ontologies, available to applications or to workflows processing high-throughput data. The service provides semantic functions that implement knowledge about the curation guidelines as well as the used relations and their interpretation. The client of the service can be embedded into custom applications and maps the service’s responses to a graph of Java objects. The OBA service provides the main information stored in ontologies to computational biologist not familiar with ontologies. The developers are enabled to concentrate on their research topic while working with the familiar object-oriented programming style.

Use cases and projects are presented to demonstrate the concept and advantages of OBA. In the use cases the Cytomer ontology and the iBeetle project are used. Cytomer^3^ is an ontology concerning anatomical structures of humans in adults and during the fetal development (Heinemeyer et al., 1999; Michael et al., 2005). Specific relations describe the progenitor, the derivation and the appearance in the Carnegie stages.

The iBeetle project^4^ aims to identify genes essential to insect development and physiology by genome wide gene silencing in the red flour beetle *Tribolium castaneum* (Schröder et al., 2008) using parental and larval RNA interference (Bucher et al., 2002; Tomoyasu and Denell, 2004). During the first part of the iBeetle project, several thousand genes have been silenced and the observed phenotypes are stored in a database and linked to an anatomical ontology for *Tribolium* (Bucher and Klinger, personal communication).

## MATERIALS AND METHODS

A service which helps to bridge the shortcomings of existing tools, as it is described in Section “Introduction,” should fulfill the following requirements:

– The service should enable an application developer to deal with the ontology in a transparent manner rather than enforcing him to deal with different ontology formats or low level APIs.
– The service should map the ontology classes and their connections to a graph consisting of Java objects.
– The part processing the ontologies should be separated from the part which is embedded in the application. A server process would in addition offer a central ontology server.
– The communication with the server should be encapsulated by a connector on the client side to provide network transparency for the custom application.
– The service should implement knowledge about the used ontologies and provide the information deduced from the ontologies by simple Java methods to a computational biologist.
– With more in-depth knowledge about the used network interface or ontologies the service should be extensible to match the requirements of new or custom ontologies and projects.

**FIGURE 1:**
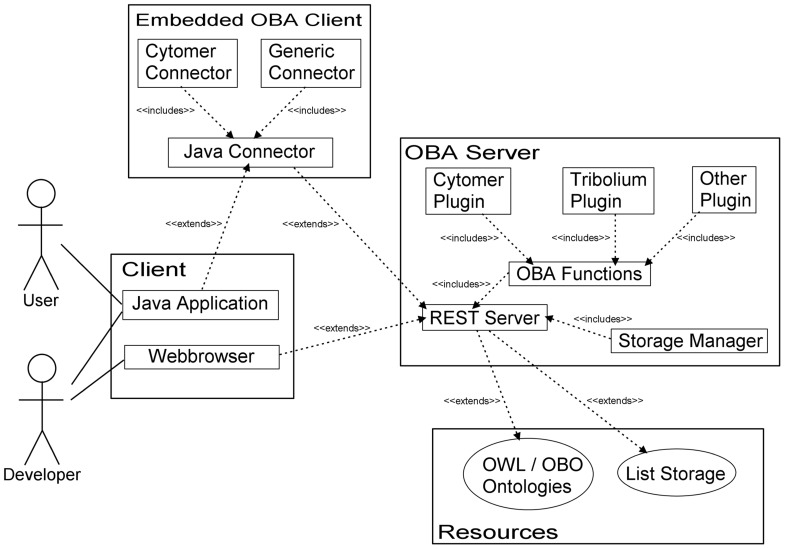
**The components of the OBA project and their relations.** The components of the OBA project are displayed together with their connections to external parts, to ontology files or to other applications.

The OBA service consists of a server and a client part, which communicate using the Representational State Transfer (REST) architecture (Fielding, 2000). **Figure 1** gives an overview of the OBA service design. The server can load any ontology in the OWL (Lacy, 2005) or OBO format (Smith et al., 2007) and host semantic functions. For every ontology a basic part of the server provides access to the entities, connected entities and lists of entities. Each entity is accessed by a unique Uniform Resource Locator (URL). Entities linked to another entity, like its child or parent classes, can also be accessed by a URL denoting the required subresource. Like the content of the ontologies, the semantic functions are available through URLs and return entities or a list of entities as answer.

A list of entities can be stored on the server in order to facilitate the work on more comprehensive input. This data can be used, for example, to limit the results of a search to members of a list of entities used in an application. To manage resource allocation, the storage area is divided into partitions. A user or a work group can create their own partition to store one or more lists. Such a partition is only accessible through its assigned name, allowing a basic access control.

The server uses a REST interface and provides the data in the “application/json,” “text/plain,” and “text/html” format (MIME-types). The open architecture allows the user to communicate with the server via a command line client, a web browser or with any custom client. The preferred form is the embedding in custom applications. For easy integration into applications a Java client is provided. The client encapsulates the network communication and facilitates access to the semantic functions of the server and to the entities of the respective ontology by Java functions. The server’s response is converted into Java objects, containing methods to access super- and subclasses as well as annotations and relations. To avoid loading the whole ontology upon the first request, the Java objects representing the ontology classes function as proxies that load connected objects upon the first access. This lazy loading is completely transparent to the application.

By default, the client uses the public server available at http://oba.sybig.de. Currently, this server provides access to the Cytomer ontology, the *Tribolium* anatomical ontology (TrOn) and the GO with ontology specific functions for the first two and generic semantic functions for all ontologies. To access custom ontologies or to implement individual OBA functions, the server and the client can be downloaded and extended. The server can load plugins to add custom OBA functions to meet new requirements of a specific project or ontology. The module containing the basic functions implements the plugin interface and can be deemed as built-in plugin. Two additional plugins, one for the Cytomer ontology and one for the iBeetle project, are already available and can serve as templates for the development of new plugins. Client extension is achieved by subclassing. These subclasses can provide Java functions to access semantic functions of a custom plugin or provide convenient functions to access annotations or relations of the ontology’s classes. Each ontology has its own defined set of relations and annotations. The generic client has no knowledge of the specific sets of annotations and relations for an ontology and enables access to the annotation and relations as two-dimensional lists containing the type of the annotation or relation and the respective values. To get the synonyms annotations of an ontology class, the application has to iterate the list of annotations until the desired one is found. A custom client can provide the method “getSynonyms()” encapsulating this iteration.

The OBA server and the example client are implemented using the Java Platform. The OWL-API is used to access ontologies in OBO or OWL format. To implement the REST-protocol the Jersey library was selected^5^. The Grizzly HTTP container handles the network communication on the server side^6^. To index the ontology’s classes the Lucene library^7^ is used. To store the metadata of the uploaded data HSQLDB^8^ was selected.

## RESULTS

With the OBA service a software application was developed to fulfill the requirements listed above (see Materials and Methods). The division into a server and a client component allows the separation of processing the ontologies from the specific custom applications. The server has access to the ontologies and hosts plugins with the OBA functions. These functions make intensive use of the ontologies and transfer the processed results to the client. The plugins encapsulate the implementation details to process the ontologies and reduce the complexity to a single function call on the client’s side. The concept of the OBA functions as a server side component is a new concept not known to the existing ontology portals.

The OBA client maps the OBA functions to Java functions and the ontology classes to Java objects. The objects representing the ontology classes have functions implemented to access their parents, children, and connected ontology classes. To avoid loading the complete ontology from the very beginning the neighboring classes are loaded upon the first access by a proxy functionality. The Java objects created by the OBA client are internally equipped with a link to the Java connector to load missing neighboring classes in the background. In contrast to existing solutions this loading process is completely transparent to the user. The developer is able to accesses the neighboring classes through Java methods and does not have to be concerned about their loading from the backend. The OBA client facilitates also access to the OBA functions by simple Java methods. Using the OBA client the network access and the implementation details of the OBA functions are transparent to the application developer, who can thus focus on the scope of his custom application.

The following use cases illustrate some OBA functions and how OBA is already used in some upcoming projects. The description of the OBA functions reveals the implementation details of these functions to show how the ontology is processed. The application developer can use these functions with a single function call and is not required to reimplement the logic.

### OBA FUNCTION: GENERIC SEARCH

The function “searchCls” is used to search for an ontology class matching a pattern that has been specified by the user. The search is not limited to the name of the ontology class, but the annotation fields of the class are included. On the client side the annotation fields to be used for the search can be specified.

**Table 1** shows the result of a search for “cistern” in the Cytomer ontology. In the second case the search is restricted to the annotation “definitionEnglish.” The search function of the Java client also provides the possibility of limiting the search to selected annotation fields. This possibility is not common in existing tools but is a powerful filter to get more precise search results.

**Table 1:** Generic search with a limitation to an annotation field.

http://oba.sybig.de/cytomer/functions/basic/searchCls/cistern	http://oba.sybig.de/cytomer/functions/basic/searchCls;field=definitionEnglish/cistern
cistern, pontocerebellar_cistern, chyle_cistern, ambient_cistern, lumbar_cistern, quadrigeminal_cistern, interpeduncular_cistern, chiasmatic_cistern, pericallosal_cistern, cistern_of_lamina_terminalis, lateral_cerebellomedullary_cistern, vein_of_cerebellomedullary_cistern, posterior_cerebellomedullary_cistern, cistern_of_lateral_cerebral_fossa, basilar_artery	pontocerebellar_cistern, basilar_artery

*Result of a search for “cistern” in the Cytomer ontology. In the first case the pattern is searched in the class name and all annotation fields including the comment field. In the right column the search is limited to the annotation “definitionEnglish” by a matrix parameter in the URL.*

The search functionality uses the name of the ontology class as well as its annotation fields and works with any loaded ontology. The classes returned by the search function can serve as starting point for traversing the graph or as input for other OBA functions.

### OBA FUNCTION: MAP ONTOLOGY CLASSES TO ANCESTORS

**FIGURE 2:**
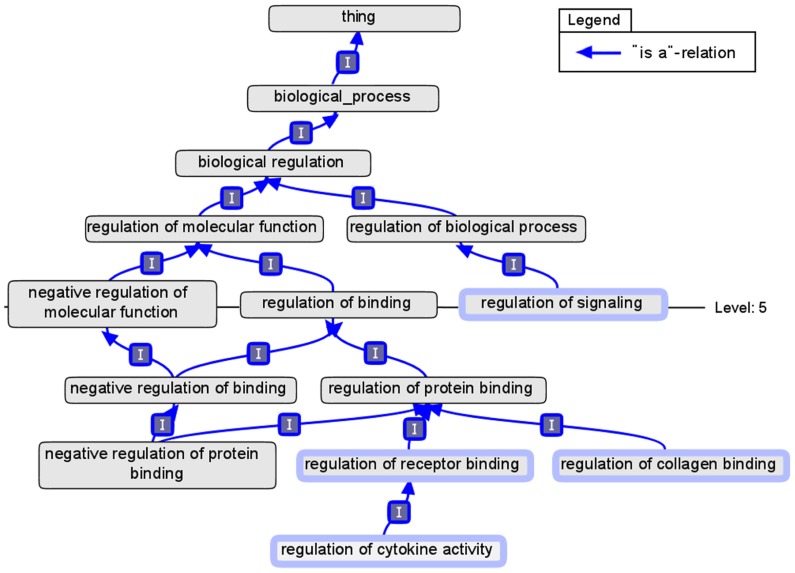
**A modified screenshot of the Gene Ontology using OBOEdit.** The node “thing” was added as root of the ontology and marks the first level. The marked nodes have a thicker border.

The goal of the following two functions is to map ontology classes to more abstract ancestors. The function “reduceToLevel” requires the input of a level and a single ontology class or a list of them. Each one of the classes from the input is mapped to all ancestors at the given level beneath the root node. To determine the ancestors of a class, all paths between the start class and the root class are considered. Due to the fact that an ontology class can have more than one parent, there might be more than one path, resulting in multiple ancestors for a single class at a specific level. If the node “negative regulation of binding” in **Figure 2** is mapped to level five, the two nodes “negative regulation of molecular function” and “regulation of binding” are returned. The function can also be called with a reference to a previously uploaded list of ontology classes. In doing so a list of classes with different levels of abstraction are mapped to classes at a constant and equal level below the root node.

A similar approach is implemented in the function “reduceToClusterSize.” In this case the ontology classes are successively mapped to their parents. In each iteration only those classes with the greatest distance to the root class are mapped to their parents. The process is finished when the number of resulting ontology classes is not larger than the specified number. The result is a list of clusters, each with a list of ontology classes from the input list, mapped to this class. Due to the specification of a maximum number of clusters instead of a concrete level, the resulting clusters may have varying distances to the root class. However, by processing the farthest ontology classes in each step, this effect is minimized. The marked nodes in the example of **Figure 2** will be mapped to the nodes “regulation of signaling” and “regulation of protein binding” if the maximum number of clusters is set to the value of two. The node “regulation of cytokine activity” is mapped in each step, while “regulation of signaling” is just copied to the result set. The classes representing the final cluster do also have different distances to the root node, five and six in this case.

The functions described in this section relay on the class hierarchy and are therefore not ontology specific, they can process any currently loaded ontology as well as the ontologies added in the future. When the described function has to be implemented with existing tools the effort is larger. To map ontology classes to a given level all classes from the starting class up to the root node have to be fetched to determine the classes on the required level. The other classes can be dismissed afterward. The OBA functions simplify the tasks by hiding the processing step behind a function call provided by the OBA client.

The result of a gene expression experiment is a list of differentially expressed genes. A common way to analyze this gene list is to map the genes to the corresponding terms of the GO. The mapping can be done for example with the help of BioMart from Ensembl (Kinsella et al., 2011). Apart from a statistical analysis the resulting list of GO terms can be mapped to more abstract terms until the list is short enough to give an overview of the main processes the GO terms belong to. This can easily be achieved with the two OBA functions “reduceToClusterSize” and “reduceToLevel” and gives a first and intuitive impression of the experiment’s outcome.

### USE CASE: CYTOMER-SPECIFIC FUNCTIONS

In the following the advantages of OBA functions provided by the service are demonstrated using the anatomical ontology Cytomer. In biomedical research different anatomical structures are investigated. These anatomical structures can be cells, tissues, organs, and entire body parts. A common example is the handling of gene or protein expression data derived from cells, organs, or biopsies (Uhlen et al., 2010). For an analysis on an equal level of abstraction, it is preferable to map all anatomical structures to the level of organs. These steps need to be automated for high-throughput data.

#### OBA function: get organs of an anatomical entity

**FIGURE 3:**
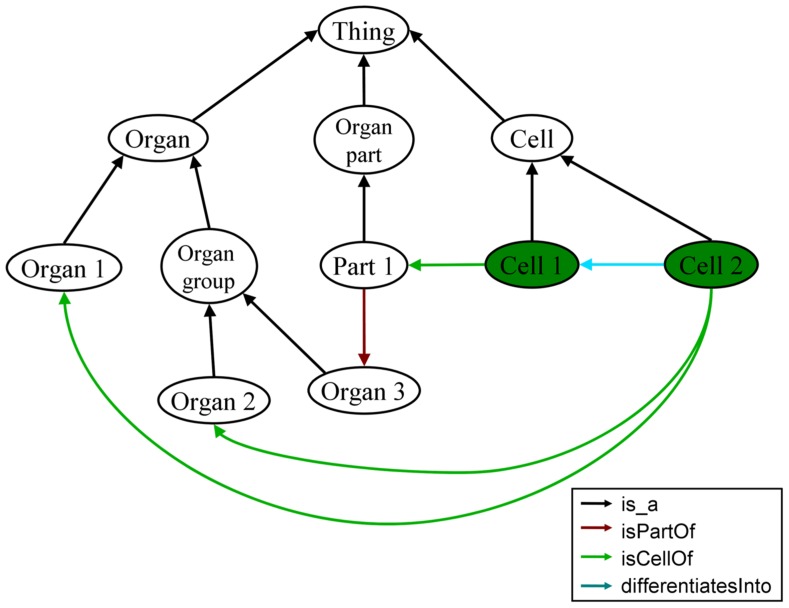
**Abstracted and simplified view of the Cytomer ontology illustrating the handling of organs of an anatomical entity.** The green nodes are the start nodes for the search function specified in the text. In this section, the entities are connected by four different relations given in the legend.

The function “organsOf” of the OBA service accepts an arbitrary class of the Cytomer ontology, which represents an anatomical structure as input and returns its respective organs. Inside this function the organs are searched along the class hierarchy and along the selected relations “isPartOf,” “isPartOfOrgan,” and “isCellOf.” Other relations, for example relations describing the development, are ignored in this case. **Figure 3** shows a simplified, abstract section of Cytomer. Using the function “organsOf” on “Cell 1” “Organ 3” is found using the two relations “isPartOf” and “isCellOf.” For “Cell 2” the two nodes “Organ 1” and “Organ 2” are found. “Organ 3” is not part of the result, because the relation “differentiatesInto” between the nodes “Cell 2” and “Cell 1” is not considered for the search of the organs of an anatomical entity. To retrieve the physiological system of an anatomical entity the function “physiologicalSystemsOf” can be used, which works in an analogous way.

### OBA FUNCTION: MAP TO A PREDEFINED LIST

**FIGURE 4:**
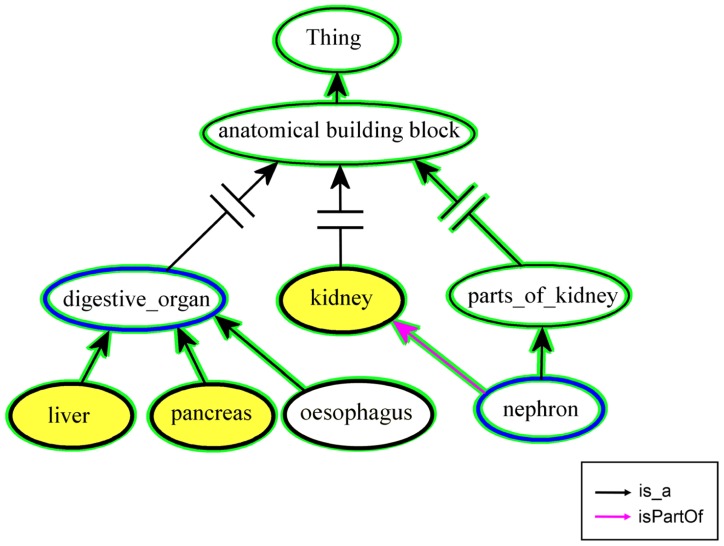
**Mapping entities to a predefined list.** The nodes with the blue border represent the start nodes for the functions “findUpstreamInSet” and “findDownstreamInSet,” respectively. The nodes and edges marked with a green background shape are processed during the mapping. The classes of the result set have to be members of the predefined set which contains the yellow nodes. A predefined list can be used by a project to limit the result of a up- or downstream search to a set of classes used in the project.

An alternative approach is to store the data linked to the most precise anatomical entities, even if these entities do not belong to the same level. In this case a user needs help to draft a request. If the request is on another level than the stored data, no match may be found, although there are relevant entries on a more abstract or more concrete level. The two functions “findUpstreamInSet” and “findDownstreamInSet” of the OBA service provide a solution for this use case. In a set-up step the list of anatomical structures represented by the input data, is stored on the OBA server. The list can be reused for each user’s request. Starting from the class, which has been requested by the user, the ontology is searched until a class in the previously uploaded set is found. For an illustration of these two functions please refer to **Figure 4**. The graph is a simplified view of the Cytomer ontology. The yellow nodes are anatomical structures used in EndoNet and uploaded to the OBA service. In the first example the user is searching information on nephron, which would give no result in EndoNet. The function “findUpStreamInSet” searches upstream of the start class “nephron,” until a class is found which is also in the previously uploaded list. In this case, following the “isPartOf” relation “kidney” is found, to which EndoNet can provide information to the user. The example of the function “findDownStreamInSet” starts with the abstract term “digestive_organ” and returns “liver” and “pancreas” as matching classes in EndoNet, by following the class hierarchy. The nodes and edges marked with a green shape are the entities processed during the mapping. The search only stops when a member of the predefined list is found, or no more nodes up- or downstream along the class hierarchy or the used relations are available.

These two functions contain a list of relations usable for the up- and downstream search. The path from the starting class to the result nodes may contain any mixture of the intended relations for the requested search direction. The length of the path is not limited, the breadth-first search stops in the iteration step with the first match and returns all matches found in this step.

The OBA functions presented above process a graph’s representation of the Cytomer ontology containing the ontology classes, the class hierarchy, and other relationships between the classes. As ontology specific information the functions have the knowledge implemented when to use which relation and how organs or physiological systems can be identified. Processing the graph’s representation is done by the OBA framework, to implement analogous functions for other ontologies or similar tasks, a new plugin can reuse this existing logic and only the ontology or task specific knowledge needs to be added, i.e., the relations to use and the key classes.

To achieve a comparable result with existing ontology portals is much more complex. In order to retrieve all organs for an arbitrary anatomical structure using the existing ontology portals the user has to decide which of the relations of the starting class could be used to traverse the ontology graph to some organ. In the next step, all neighboring classes linked by the selected relations have to be queried from the portal. The last two steps have to be repeated for every fetched intermediate class multiplying the number of classes in each step. Whether one of the processed classes represents an organ has to be decided by the users based on their medical knowledge or based on rules deduced from the curation guideline of the ontology. Using the OBA function “organsOf” all these steps are executed on the server where the knowledge is implemented which ontology classes represent the concrete organs. Due to the multitude of relations to consider, 70 ontology classes are processed to return “liver” as organ for the ontology class “hepatocyte.” To get the organs lung, larynx, and trachea for the ontology class “sensory_epithelial_cell” 2,497classes are needed to be checked. Without OBA each of these classes has to be downloaded from an ontology portal and processed locally. The numbers are dependent on the starting class and the version of the used ontology. New or removed relations can have a great impact on the number of processed ontology classes. However, for simple queries like the example of the hepatocyte cell, a considerable number of ontology classes already have to be processed. Using OBA the result is always achievable with one single function call. Even changes in the annotation guidelines, like new relations’ types, of the used ontology would be encapsulated in the plugin and hidden from the application developer.

### PROJECT: iBeetle

In the iBeetle project genes are silenced by RNAi and the observed phenotypes for several stages are annotated into a database following the Entity–Quality (EQ) system (Washington et al., 2009). During the project a detailed ontology about the anatomical structures of *Tribolium *in different developmental stages has been created. There is an ontology class for each structure at every developmental stage where this structure exists. Thus there are distinguished classes for the pupal and the larval antenna. Both are linked with an “isPartOf” relation to the corresponding developmental stages and share the same generic superclass “antenna”. The annotations are linked to the classes connected to a developmental stage instead of being linked to generic ones. The most detailed level in the ontology is chosen for the annotation, i.e., flagellum is used if the phenotype affects only the flagellum and not the whole antenna. For the search interface the requirements are different. A typical input is the developmental stage and a generic and rather abstract morphological structure, e.g., antenna instead of flagellum. To fulfill the demands and provide a general access to the *Tribolium* ontology the OBA service is embedded into the search interface and a server plugin with specific semantic functions has been implemented.

**FIGURE 5:**
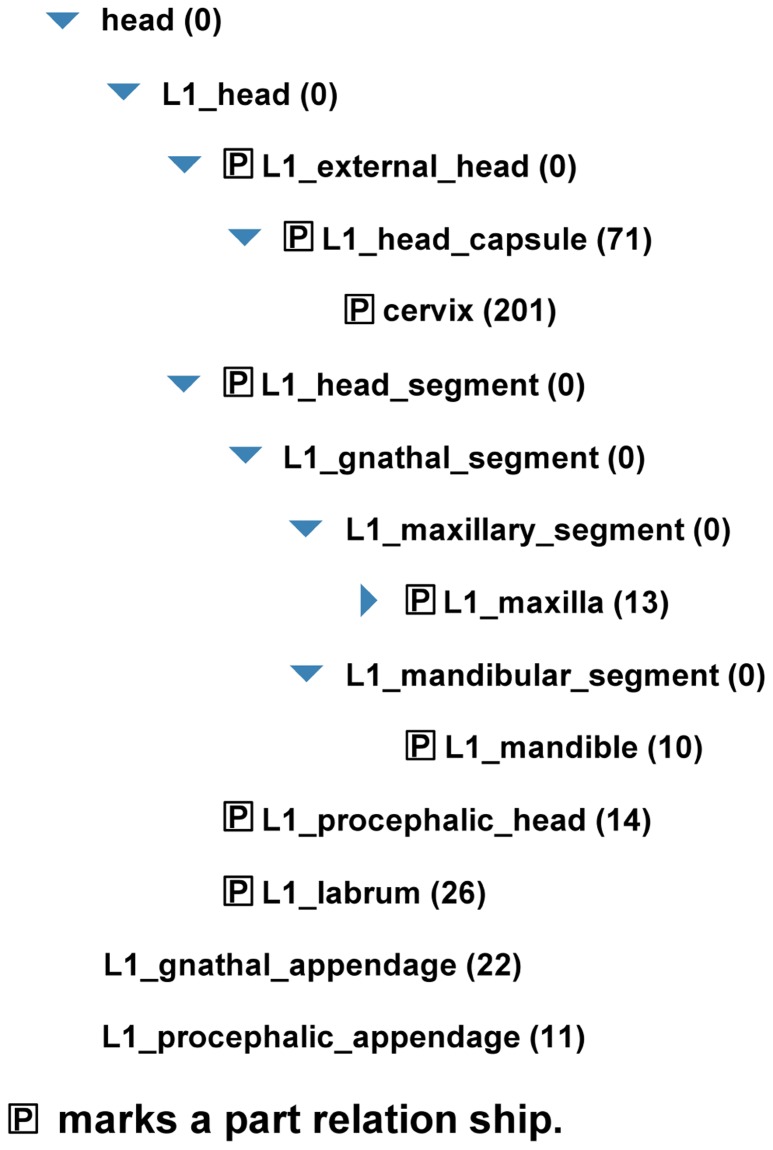
**Ontology tree from the result page of a search in the iBeetle database.**The tree shows the classes of the *Tribolium* ontology downstream of the searched structure and linked to the queried developmental stage. In this example, the user has selected “head” as anatomical structure and “larva” as stage. All ontology classes shown in the tree where used for a search in the iBeetle database. The numbers in parentheses indicate the number of hits linked to this node.

Upon startup the OBA service scans the ontology for concrete classes (these connected to a developmental stage) and generic classes, respectively. The concrete classes do not necessarily have a direct relation to a developmental stage, the path to the stage may be a collection of “is_a” and “isPartOf” links. The generated list of generic classes is used as a suggestion list for the user while typing into the search form. When the user has chosen a developmental stage and an anatomical structure, the OBA service selects all concrete classes downstream of the selected structures and connected to the appropriate stage. Because “isPartOf” is used in the *Tribolium* ontology to describe meronomic relation, the inverse “hasPart” relation is generated on the fly. The list of ontology classes is used as input for the search in the database of the iBeetle project. As add-on on the result page a tree with the subsections of the ontology that were used for the search is displayed. **Figure 5** shows a screenshot of this ontology tree. The semantic search started with the search term “head” and added all ontology classes representing head and its parts.

### PROJECT: EndoNet

For the upcoming new web interface for EndoNet, an information resource of the human endocrine system (Dönitz et al., 2008), a semantic search, similar to the search function described above is used. As ontological data source the anatomical ontology Cytomer is utilized. In this case the focus is not on developmental stages but on grouping the annotated cells and tissues at the level of organs in order to generate a survey map of general pathways. To limit the search result to anatomical structures used in EndoNet a predefined list containing the anatomical structures used in EndoNet is stored on the OBA server.

### PROJECT: OntoScope

**FIGURE 6:**
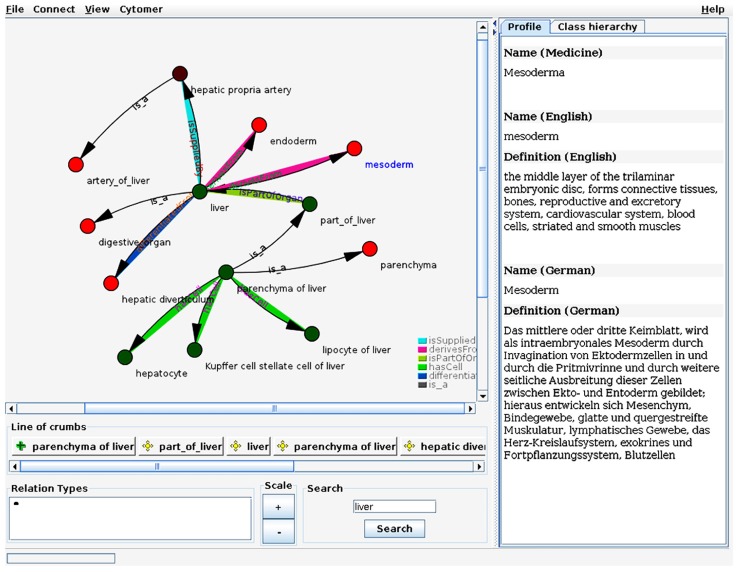
**Screenshot of OntoScope.** The ontology viewer OntoScope uses the object graph from OBA to let the user browse the graph. The ontology classes are represented by the nodes of the graph. Each relation type has an own color and are displayed as edges connecting the nodes. The color of the nodes indicates the physiological system the class belongs to and is retrieved from the OBA service. On the tabs of the right side additional information of the selected ontology class is displayed, or the class hierarchy of the classes in the graph (tab is hidden in the screenshot).

Another type of application using the OBA service is the ontology viewer OntoScope^9^. OntoScope visualizes ontologies as a graph extending the common tree like view of ontologies. The representation as a graph enables the user to explore ontologies along arbitrary relations. OntoScope uses from the OBA service the object graph and the access to the ontologies without any knowledge about the format or semantics of the ontology. OBA functions are used in the background, so that for example the nodes of the Cytomer ontology can be displayed in a color code according to the physiological system. **Figure 6** shows a screenshot of OntoScope with several nodes and relations.

**Table 2** summarizes the OBA functions used in the projects. The plugin containing the function is named and a short description is given.

**Table 2:** Overview of the OBA functions used in the projects.

Project	Used OBA function	Plugin	Functionality
iBeetle	concreteClasses	Tribolium	Returns all classes linked to a developmental stage. The annotated phenotypes are linked to these classes
	genericClasses	Tribolum	Returns all classes not related to a developmental stage, used for the auto-complete function of the search interface
	findInGeneric	Tribolium	Searches in the labels and synonyms of generic classes and an additional previous generated list for classes matching the search string. Used for the auto-complete function in the search interface
	concreteForDevStage	Tribolium	Returns the class downstream of the given generic class and linked to the given developmental stage. Used to map the user query to the annotations stored in the database.
EndoNet	findUpStreamInSet findDownStreamInSet	Cytomer	Used to find entities from EndoNet related to the search term
OntoScope	physiologicalSystemOf	Cytomer	Returns all physiological systems of an ontology class, used for coloring in the graph
	searchCls	built-in	Searches ontology classes matching a text pattern in the class name or annotation field

*The table summarizes the use of OBA in the projects listed in the first column. The second and the third column denominate the OBA function name and the plugin containing the function. The last column describes the functionality of the OBA service the projects benefits from.
*

### INSTALLATION AND EXTENSION OF OBA

For the use of OBA in a new application the Java client has to be downloaded and added to the class path of the application. After the initialization of the connector, all OBA functions are accessible as Java methods through the connector. The OBA functions will return single ontology classes or lists of them. These ontology classes are mapped to Java objects by the connector and returned by the Java methods of the connector. The Java objects provide functions to access the annotations and neighboring classes of the represented ontology class. If necessary missing information is queried internally from the OBA server. The application developer does not have to be concerned about the retrieval of neighboring classes.

If a required ontology is not available on the public OBA server, it can be downloaded and started locally. After the extraction of the zip file default directories for ontologies, plugins, and the storage area are available. New ontologies can be copied to the ontology directory together with a short property file. The property file defines under which name the ontology will be available from the OBA server and which annotation fields should be indexed for the search function. The property file can be copied from the provided examples and is described in the manual.

### SUMMARY

The OBA service is available online at http://oba.sybig.de. Upon pointing a web browser to this URL an overview is given as a list of loaded ontologies as well as the available plugins and the OBA functions implemented by them. The object graph of the ontologies can be browsed by following the links of the HTML representation of the ontology classes. The syntax to access the OBA functions is described in the manual available at the home page of the project: http://www.bioinf.med.uni-goettingen.de/projects/oba. Located on the home page of the project is the Java connector as well as all sources and jar files for the server and currently available plugins. The Cytomer connector contains a test client, which is executed when the client is run on the command line. This client calls some functions on the server and prints the results to the console in order to validate the OBA service’s function. The client’s sources can serve as a template for a usage of OBA in a custom application.

To give the user a first impression of the function of the OBA service, a web demo is available at http://webdemo.oba.sybig.de/ implementing some of the provided functions for manual tests. For each step the example source code is noted, which is needed to implement the corresponding step in a custom application.

## DISCUSSION

Ontologies are powerful and also complex tools. This is especially true for the OWL format. Parsers like the Jena-API (Jena – A Semantic Web Framework for Java^10^) or the OWL-API (Horridge and Bechhofer, 2011), take care of parsing ontologies but do not intend to hide the semantics of ontologies. The same is true for OBO ontologies, although they have a more finite structure. If a developer plans to include information deduced from ontologies in an application, a time for training is needed to learn the semantics of ontologies and the framework’s design. The basic tutorial of the OWL-API already consists of over 100 slides and deals with a semantic most computational biologists are unfamiliar with. The OBA service maps the relevant parts of ontologies to the world of object-oriented programming and provides semantic functions. The usage of the OBA service does not call for intensive training time to work with different topics and programming paradigms. The simplification to an object graph is oblivious to advanced features of OWL like cardinalities or different OWL dialects. If such a full access is needed, it can be achieved with the very good ontology APIs, i.e., Jena-API or OWL-API, with the query language SPARQL or Protege for interactive work. However, the OBA service can load and process any ontology in the OBO or OWL format, giving access to their fundamental information to developers who otherwise would probably not use ontologies.

Portals like OntoCAT (Adamusiak et al., 2011), the OLS (Côté et al., 2008), or the NCBO BioPortal (Noy et al., 2009) aim to provide access to huge collections of ontologies in a standardized manner. This is the preferred way if the unique definitions of terms in ontologies take precedence over the complex relations. Like the OBA service, OntoCAT and the NCBO ontology portal allow the user to access ontologies using the REST-protocol. OntoCAT also provides basic clients for different programming languages. In addition to the functions of the OntoCAT client, the Java objects of the OBA service provide the required functions to access the super- and subclasses as well as classes which are linked by relations. Together with the proxy function, the basis of the new feature in the OBA service is to map ontology classes to an object graph, traversable by Java methods. The required network communication with the service is encapsulated by the OBA client and transparent to the user. The feature to grant access to the neighbors of an object, representing an ontology class, by Java methods is beyond the function provided by the clients of the existing ontology portals. Together with the proxy function of the OBA client the developer is now enabled to access ontology classes and traverse the graph using only Java methods. Network access and parsing of the ontology is transparent.

One intention of the OBA service is to relieve the user from ontology specific demands by encapsulating the logic in a service. With the OBA functions the developer benefits from the rich information of a specific ontology encoded in the relations without the detailed knowledge about these semantics. The goal of the OBA service is not primarily to provide network access to ontologies, but to add additional functions to help a developer to solve a subtask of an application based on information available in ontologies without being familiar with ontologies, APIs, or query languages to process them.

The OBA service’s concept of semantic functions is distinct from the goal of ontology portals like OBO-Foundry (Smith et al., 2007), NCBI, or OntoCAT. The portals focus on accessing as many ontologies as possible. This approach is very well suited for an ontology overarching search and access. The OBA service provides access to a set of specific ontologies with matching semantic functions. If a plugin with the required semantic function is already available the developer saves time for training and programming. Even if the required function is not available, the developer benefits from the framework of the OBA service and the advantages of the client described above. The OBA framework and the open architecture minimize the effort of extending the service to fit the requirements of a specific project. A new plugin relays on the existing functions to access the ontology, marshal the objects for the network transfer as well as the proxy functionality of the client. A new plugin only has to implement knowledge about a custom ontology or the logic to solve a new question. Due to the provided framework the already supplied plugins are very small and easy to implement. The developer of a new plugin needs to be familiar with the curation guideline of the used ontologies. Further expertise about ontologies, like the different formats and ontology internals like Frames, Description Logic are not required.

Under the umbrella of the OBO-Foundry a collection of tools handling ontologies has evolved. There is a number of tools supporting the annotation process or focusing on statistical analysis of data based on ontologies, examples are the tool DAVID (Huang et al., 2009) and tools for the gene set enrichment analysis (GSEA) method (Subramanian et al., 2005). Like the functions of the OBA service, these tools make intensive use of the GO or other ontologies. The advantage of the OBA service is that it is easily extendible. The server can load plugins for any ontology. The service is designed to be embedded into applications and workflows to minimize interaction with external tools.

The design of the OBA service has several advantages. A public server is the central contact point and serves a growing collection of publicly available ontologies and plugins. Developers and maintainers of an ontology are welcome to submit new plugins, which enables the scientific community to profit. Alternatively, the server can be downloaded and run locally if the required ontology is not available in the public repositories, or if the developed plugin is not to be published.

The new features of OBA are the seamless mapping of ontologies to a connected object graph for object-oriented programming and the implementation of the OBA functions.

The server side plugins can make intensive use of the ontologies loaded by the server and return the computed results back to the client. The round-trips between client and server are reduced to a minimum and the logic is encapsulated in a reusable plugin. This new features enables computational biologists to use the basic information from ontologies in their applications, who would otherwise avoid ontologies.

## Conflict of Interest Statement

The authors declare that the research was conducted in the absence of any commercial or financial relationships that could be construed as a potential conflict of interest.

## Footnotes

1 http://protege.stanford.edu

2 http://jena.sourceforge.net

3 http://cytomer.bioinf.med.uni-goettingen.de

4 http://ibeetle-base.uni-goettingen.de/

5 http://jersey.java.net/

6 http://grizzly.java.net/

7 http://lucene.apache.org/

8 http://hsqldb.org/

9 http://www.bioinf.med.uni-goettingen.de/projects/ontoscope/

10 http://jena.sourceforge.net
